# Efficient Surrogate Modeling and Design Optimization of Compact Integrated On-Chip Inductors Based on Multi-Fidelity EM Simulation Models

**DOI:** 10.3390/mi12111341

**Published:** 2021-10-30

**Authors:** Piotr Kurgan

**Affiliations:** Department of Microelectronic Systems, Faculty of Electronics, Telecommunications and Informatics, Gdańsk University of Technology, Narutowicza 11/12, 80-233 Gdansk, Poland; piotr.kurgan@pg.edu.pl

**Keywords:** integrated inductors, electromagnetic simulation, surrogate modeling, design optimization, simulation-driven design, space mapping

## Abstract

High-performance and small-size on-chip inductors play a critical role in contemporary radio-frequency integrated circuits. This work presents a reliable surrogate modeling technique combining low-fidelity EM simulation models, response surface approximations based on kriging interpolation, and space mapping technology. The reported method is useful for the development of broadband and highly accurate data-driven models of integrated inductors within a practical timeframe, especially in terms of the computational expense of training data acquisition. Application of the constructed surrogate model for rapid design optimization of a compact on-chip inductor is demonstrated. The optimized EM-validated design solution can be reached at a low computational cost, which is a considerable improvement over existing approaches. In addition, this work provides a description and illustrates the usefulness of a multi-fidelity design optimization method incorporating EM computational models of graduated complexity and local polynomial approximations managed by an output space mapping optimization framework. As shown by the application example, the final design solution is obtained at the cost of a few high-fidelity EM simulations of a small-size integrated coil. A supplementary description of variable-fidelity EM computational models and a trade-off between model accuracy and its processing time complements the work.

## 1. Introduction

On-chip inductors are essential components of various radio frequency integrated circuits (RFICs), including low-noise or power amplifiers [1,2], mixers and voltage-controlled oscillators [3,4]. One of the key factors that determine the performance of the above-listed RFICs is the quality factor of the implemented inductors [5]. For available silicon-based technologies, this parameter is considerably limited by fundamental energy dissipation mechanisms related to high metal resistivity as well as coupling with the conductive substrate [6]. A popular solution to this problem is to utilize a patterned ground shielding that decouples the inductor from the silicon substrate [7]. A different approach is to capitalize on thick metals, low-permittivity package dielectrics, and a larger distance from the lossy substrate by implementing the inductor above the chip passivation using the redistribution layer of a wafer-level package [8]. Apart from that, another important design aspect to consider is to obtain the necessary electrical parameters of the system while keeping the area occupied by the inductor as small as possible. This is essential from the perspective of fabrication cost reduction, as on-chip inductors are known to consume a significant part of the chip area [5]. However, regardless of the applied circuit or packaging solutions, the measured figures of merit of a fabricated coil (including the quality factor) typically violate design specifications or illustrate inferior values in comparison to the predicted data, which is due to insufficiently accurate design tools involved in the design process. In the case of circuit realizations whose key performance parameters lie beyond the tolerance threshold, the entire design cycle must be repeated. Thus, to eliminate the risk of design failure, it is imperative to ensure the highest standards of design reliability. Precise characterization of a given inductor can only be obtained through high-fidelity electromagnetic (EM) analysis, which takes into consideration a variety of complex and interrelated physical phenomena such as oxide and fringing capacitances, the skin effect, current crowding, eddy currents, or resistive losses [9]. Moreover, EM solvers are capable of effectively handling structures of arbitrary geometry and accounting for the proximity of periphery elements (e.g., soldering ball or keep-out zone). The main downside, however, is that the accurate EM simulations are typically very CPU-intensive, which makes their repetitive use in any design procedure involving multiple objective function calls (e.g., exhaustive enumeration or optimization routines) an extremely challenging task. For this reason, the high-fidelity EM simulations are used merely as a support for less time-demanding design tools (e.g., for parameter extraction of lumped-element circuit models [10]) or for design verification [11]. Design tools used extensively in practical RF/microwave engineering incorporate suitable simplifications of the EM problem at hand that normally lead to less accurate results but within a reasonable timeframe. One example is reducing the cost of the original calculations by means of electrostatic and magnetostatic approximations [12]. However, the primary disadvantage of this technique is inadequate modeling of eddy currents induced in the substrate, which translates into the poor assessment of the quality factor of an RF inductor [13]. The more prevalent approach is to use circuit theory to model the frequency behavior of integrated coils [14]. Advanced lumped-element models often exhibit limited generalization capabilities, due to frequency-independent parameter values [9] as well as single-point parameter extraction procedures [10]. To this date, several different methods have been used for the design optimization of integrated inductors represented by simplified physics-based models listed above. These include geometric programming [15], sequential quadratic programming [16], mesh-adaptive direct search [17], and genetic algorithms [11]. Despite their computational superiority over an exhaustive enumeration, the aforementioned techniques are incapable of handling computationally-intensive inductor models based on full-wave EM solvers.

Design difficulties related to the excessive numerical cost of direct EM optimization can be alleviated to some extent by exploring the surrogate-based optimization (SBO) [18] concept. In general, SBO combines the computational efficiency of low-fidelity models (e.g., equivalent circuits) with the accuracy of high-fidelity EM simulations. The SBO optimization algorithm performs the bulk of numerical operations on a cheap model while referring to the expensive EM solver only occasionally to calibrate the low-fidelity model or to verify the approximate design solution. Typically, a satisfactory design is acquired using a handful of EM analyses. The initial attempt to adopt the SBO technology for design optimization of planar inductors [19] involved the utilization of implicit space mapping (ISM) [20] and compact equivalent circuits as an underlying low-fidelity model. The exploitation of space mapping in the process of rapid and reliable design of spiral inductors exhibits great potential, although problem formulation used in this work—based on geometric programming—may significantly limit the applicability of the method for other, more complex inductor geometries. In addition, the effectiveness of space mapping strongly depends on the correlation between low- and high-fidelity models [18], which is typically insufficient in the case of lumped-element circuits.

The present work reports on further developments concerning the application of SBO to reliable modeling and expedited design optimization of integrated on-chip inductors. Instead of relying on geometry-specific equivalent circuit models that offer poor prediction capabilities, the author provides means to develop fast and well-correlated kriging interpolation models based on sampled coarse-discretization EM simulations. The ultimate model correction—elevating it to the level of a high-fidelity EM model—is realized through a combination of input, frequency, and multiplicative output space mapping [21,22]. The optimal EM-validated design solution can be found in a few iterations of the presented SBO algorithm. As a viable alternative, the author adapts a variable-fidelity optimization algorithm to perform rapid design optimization of a small-size on-chip inductor. The procedure incorporates a family of EM models with a gradually increasing complexity that are optimized in succession to produce—in a cost-efficient manner—intermediate designs tending towards the high-fidelity optimum. The final design solution obtained this way is subsequently refined by an SBO routine using a local polynomial approximation model and output space mapping to attain the accuracy of a high-fidelity EM model. To the best of the author’s knowledge, both design strategies presented in this work outclass other previously reported EM-driven methodologies relevant here in terms of computational efficiency. In addition—unlike prior methods—the techniques under consideration are not limited to standard and well-established inductor geometries and are methodologically more convenient, making use of one simulation tool instead of two.

## 2. Integrated On-Chip Inductors: Design Challenges and Simulation Models

In this section, a brief introduction to the EM-driven design of planar spiral inductors for RFICs is presented, including revision of standard coil structures, investigation of inductor EM simulation models of graduated complexity as well as a description of the common design challenges.

### 2.1. Integrated On-Chip Inductors: Typical Structures and Applications

On-chip inductors are fundamental components of modern RFICs, finding diverse applications in highly integrated RF transceivers as building blocks of inductance-capacitance tank oscillators, mixers, low-noise amplifiers, filters, or matching circuits [23]. Continuous efforts are made to obtain high-performance and area-efficient implementations of on-chip inductors on standard silicon substrates. This is particularly important because the quality factor of on-chip inductors—typically ranging from 10 to 15 [8]—is a major performance-limiting factor of RFICs in terms of power consumption, power efficiency, and phase noise level [24]. For this reason, improvement of the quality factor of RF integrated inductors is the prevailing design objective in the relevant literature, besides attempts directed towards ensuring compact inductor layouts.

**Figure 1:**
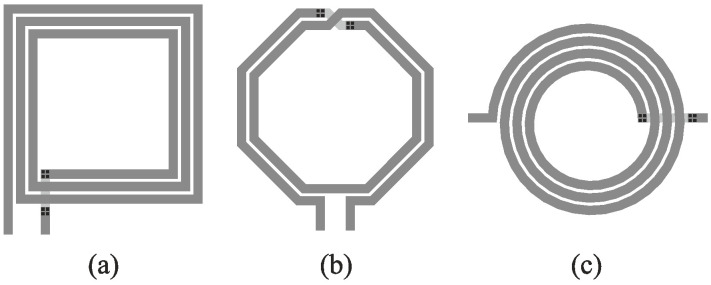
Typical on-chip inductors: (**a**) rectangular coil in single-ended configuration, (**b**) octagonal coil of differential type, and (**c**) circular coil in unbalanced configuration. Windings, undercrossing, and vias are represented in the picture by dark gray, light gray, and black colors, respectively.

On-chip inductors may be implemented in a variety of ways (e.g., as a straight line, toroid, or solenoid to name a few), but most commonly they are realized in the form of a spiral [5]. For this popular type of integrated coil, a metal track is routed around the center point of the structure, and—depending on the angles between the bending conductor, permitted by a given fabrication technology—the geometry of the coil may be rectangular, octagonal, circular, etc. Another important feature of an inductor is its configuration type. We distinguish between unbalanced (single-ended) or balanced (differential) configurations. The latter is typically required for differential applications, where the goal is to minimize common-mode interference [5]. Figure 1 depicts standard structures of on-chip inductors with spiral geometry.

### 2.2. Computational Models of Integrated Inductors

**Figure 2:**
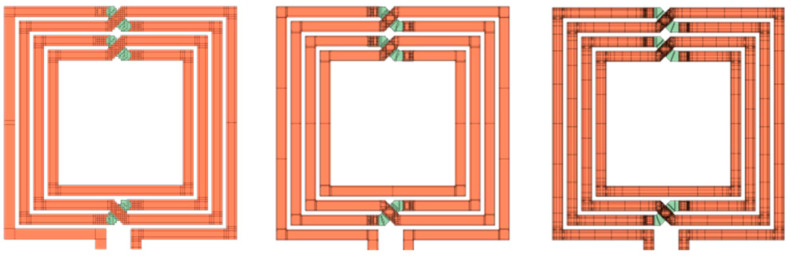
Examples of EM models of graduated complexity of a rectangular 4-turn, 1-layer coil. From left to right: coarse model without edge meshing, routed on a 2 × 2 grid; coarse model with edge meshing on a 1 × 1 grid, and fine model created on a 0.5 × 0.5 grid. Grid dimensions are in microns.

Today, the prevailing approach to modeling integrated inductors is primarily based on equivalent circuit models, whose simplified description cannot precisely account for complex and interrelated high-frequency phenomena that occur with respect to physically integrated inductors. On the other hand, high-fidelity computational models offer definite accuracy, but their sole use in simulation-driven design methodologies is greatly limited by the overall numerical cost. To address the above-outlined issues, some SBO techniques exploit computational models of lower fidelity [25]. These are still well correlated with their high-fidelity counterpart but are far less CPU intensive. In addition, variable-fidelity models do not require any auxiliary simulation tools, as they are implemented in the same simulator as the high-fidelity model. Typically, they are obtained by applying relaxed mesh settings that affect the discretization rate of the structure under consideration. Numerous alternative ways of reducing the complexity of the simulation model—often combined with decreasing mesh density—are commonly available, including using a perfect conductor of infinitesimal thickness instead of thick copper, incorporating lossless and isotropic dielectric substrate in place of lossy and anisotropic one, reducing computational domain, inserting discrete source as a substitute for a waveguide port, etc. Examples of variable-fidelity EM models of a planar inductor are shown in Figure 2.

In practice, the number of mesh cells of the discretized structure at hand is directly controlled by solver-specific settings. For example, in a commercial EM software package, Sonnet ***em***, this can be accomplished through the grid, meshing, and metal model settings [26]. Sonnet ***em*** incorporates a surface meshing technique so that only the circuit metallization is sub-sectioned to the accuracy of the uniform fixed grid. In that regard, individual subsections cannot be smaller than the underlying grid itself, yet their exact size and placement are controlled by choosing from one of three meshing options. Fine meshing provides the highest accuracy but also demands the largest memory and processing time. The second option produces mesh on the edges of the structure and generates coarse subsections in the remaining parts of the circuit, which results in a good compromise between the speed and accuracy of EM analysis. The last option yields the least accurate results as the entire circuit is divided into large subsections, without distinguishing critical parts of the structure (e.g., edges). Finally, metal model settings enable modeling of thick metals by adding additional shunted metal layers to the original polygon, which allows for proper consideration of the coupling effects between closely separated conductors (e.g., interwinding capacitance).

**Figure 3:**
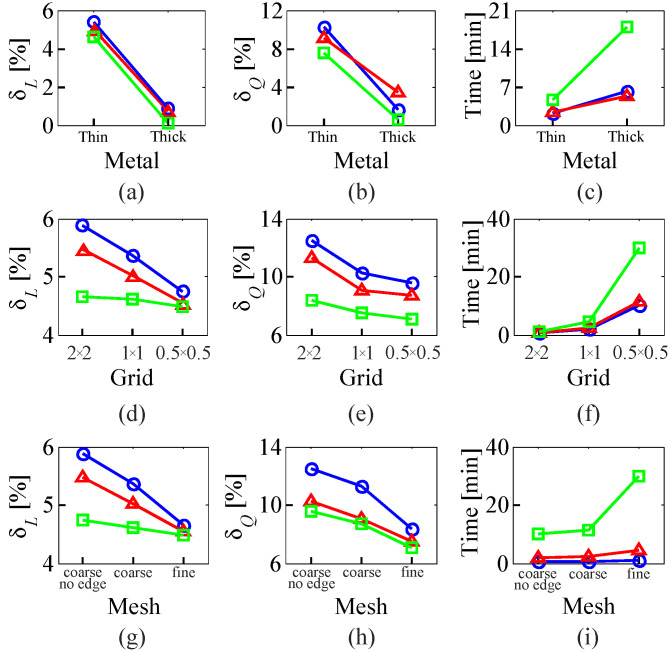
Comparative analysis of variable-fidelity EM models of a rectangular 4-turn upper-layer inductor realized in a differential configuration (cf. Figure 2). Key model performance parameters are: inductance approximation error (*δ_L_*), quality factor approximation error (*δ_Q_*)—both calculated for the peak frequency—and simulation time. Figures (**a**–**c**) showcase simulation results for EM models with grid fixed to 1 × 1 (in microns) and different mesh settings: coarse (no edge) mesh (○), coarse mesh (△), and fine mesh (Δ). Figures (**d**–**f**) present simulation data obtained for models with a fixed thin metal model. Models with different mesh settings are marked identically as for Figures (**a**–**c**). Figures (**g**–**i**) compare EM models whose metal is approximated by a thin model. Different curves denote different grid settings: 2 × 2 (○), 1 × 1 (Δ), and 0.5 × 0.5 (□)–all dimensions in microns.

Following the above-listed guidelines, the high-fidelity EM model of an on-chip inductor is developed in Sonnet ***em*** by using a fine grid, fine meshing and thick metal model for upper-layer winding. It should be reiterated that design optimization based exclusively on such a model is often prohibitive. To alleviate this problem, EM models of lower fidelity are obtained by compromising model accuracy with respect to its grid, meshing, and type of metal model. However, the influence of lesser model settings on its processing time and accuracy is not obvious and requires investigation. The latter was accomplished here by analyzing simulation results of a family of variable-fidelity EM models representing all available combinations of accuracy settings. The assessment of model speed and accuracy was conducted based on the simulation time as well as inductance and quality factor approximation errors (calculated using the relevant quantities of the high-fidelity model). The selected results of variable-fidelity EM model comparison are presented in Figure 3. The inspection of Figure 3 reveals the independent metal model, grid, and meshing effects for different EM model settings.

As indicated by the results collected in Figure 3, the relaxation of specific model settings results in a dramatic CPU cost reduction, which translates into a shorter evaluation time. Acceleration of EM simulations is accomplished at the cost of lesser accuracy—up to 14% of the quality factor approximation error, considering the degradation of only one model feature. Simultaneous relaxation of all model settings typically results in unacceptable error accumulation. In general, it is not clear how to find a suitable trade-off between model accuracy and its processing time. The rule of thumb is that the model simulation time should be as small as possible, provided that the performance of the lower fidelity model preserves the key features of the high-fidelity EM model (e.g., the shape of frequency characteristics, including an approximate placement of resonances, etc.). The latter condition is essential for the proper operation of SBO algorithms [25].

### 2.3. Design Challenges

The reliable design of spiral inductors for modern RFIC applications is a challenging task that involves simultaneous adjustment of multiple designable layout parameters of a given structure to satisfy several objectives. Meeting the specified design requirements with high precision at a relatively low computational cost is a fundamental prerequisite of the entire design process. A typical design problem of an integrated coil is to obtain a target inductance value as well as the highest quality factor at the desired operational frequency. Another important design criterion is inductor size minimization or fitting it inside a restricted die area.

Design optimization of a spiral inductor is usually preceded by establishing details of its geometry (e.g., shape, configuration, number of metal layers, etc.). This is accomplished based on information on the available technology process and the specific application. For example, reducing the series resistance of a given inductor—which is imperative for the improvement of the quality factor—can be achieved by using upper-layer thick metals for winding [27]. A similar effect is obtained by shunting metal layers to form a parallel-stacked inductor [28]. The limiting factor of the latter is associated with additional resistance of the interconnecting vias and increased resistance of the lower-layer thin undercrossing. For more detailed design guidelines, the interested reader is referred to the literature (see, e.g., [11]).

Once the inductor topology is determined, the desired circuit performance is achieved by means of design optimization. In the literature, this is typically carried out by maximizing the quality factor, subject to an equality constraint concerning the target inductance. An alternative approach is based on sequential parameter sweeping guided by engineering insight, where each parameter is treated separately [29]. The above techniques exhibit considerable limitations—the former method is commonly used in combination with inaccurate equivalent circuit models, which leads to poor results when validated by an EM simulation; the latter neglects interrelations between designable parameters, which results in sub-optimal design solutions.

This work presents reliable and cost-efficient design optimization methodologies dedicated to computationally demanding models of integrated inductors. Contrary to the previously reported methods, the proposed techniques do not rely on equivalent circuits of limited accuracy but exploit a combination of variable-fidelity EM models and space-mapping-corrected data-driven models to arrive at the optimal design solution using only a handful of high-fidelity EM simulations.

## 3. Efficient Surrogate Modeling and Design Optimization

This section presents a reliable surrogate modeling of planar inductors implemented in standard CMOS technology. The main components of the reported modeling technique include low-fidelity EM simulations, kriging interpolation, and space mapping as means of model enhancement. The developed surrogate model is very accurate over a wide frequency bandwidth. As an application example, reliable and expedited design optimization of a planar on-chip inductor with a compact layout is provided.

### 3.1. Design Case

**Figure 4:**
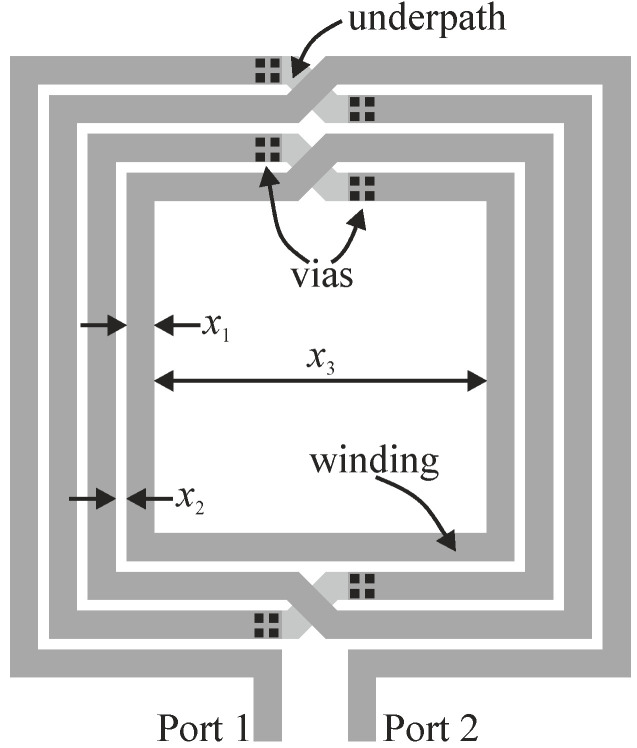
Layout of the four-turn integrated on-chip inductor.

**Figure 5:**
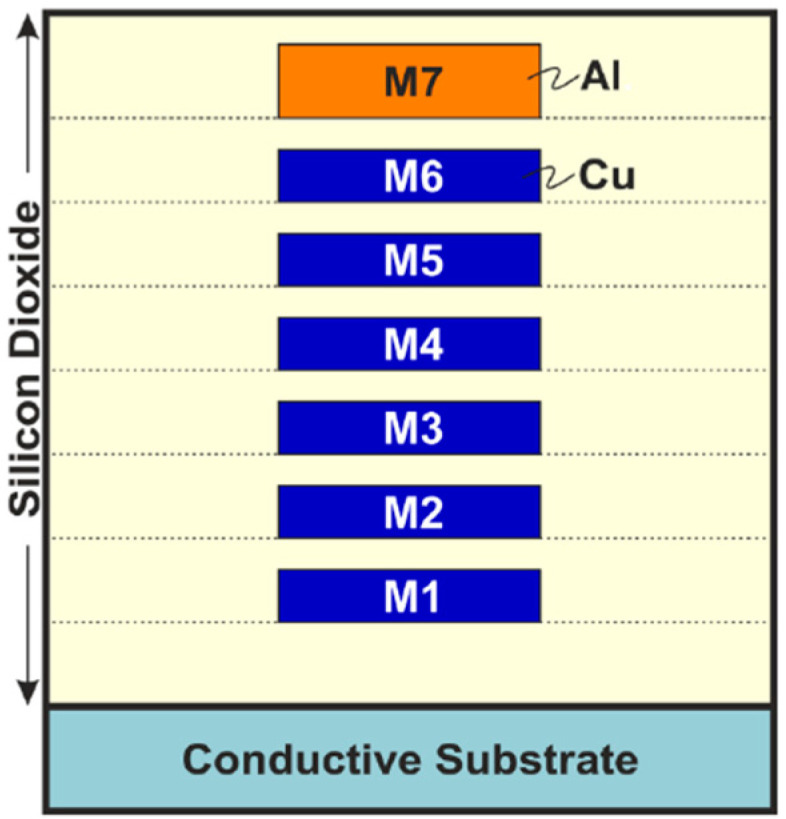
The 65-nm CMOS technology stack used in this work. Inductor windings are routed using M7 metal layer, whereas layer M6 is used for undercrossing (M7 and M6 are connected with vias).

Consider a structure of an on-chip inductor shown in Figure 4. The device is realized in a standard 65-nm CMOS technology process that offers six thin copper layers and a single 1.35-μm thick aluminum layer placed on the top of the stack. Metal layers are embedded in silicon dioxide and reside on a conductive substrate (cf. Figure 5). The presented coil is designed in a differential configuration with a thick, four-turn top-layer aluminum winding and a thin copper undercrossing. The designable parameters of the inductor, given by the vector ***x*** = [*x*_1_ *x*_2_ *x*_3_]*^T^*, include track width, track-to-track distance, and inner radius, respectively.

The primary objective is to develop a highly accurate broadband model of the structure under consideration. This is accomplished in Section 3.2 by using low-fidelity EM simulations, response surface approximation, and space mapping technology for model refinement [30].

### 3.2. Surrogate Modeling Using Response Surface Approximation and Space Mapping

The modeling technique presented in this work is based upon the concept of surrogate modeling and combines EM simulations of low fidelity, response surface approximations, and space mapping [18,21,22].

The primary component of the proposed method is a low-fidelity EM model of the considered inductor. The reason for using such a model instead of a high-fidelity one is that the latter is normally too CPU-intensive to be extensively sampled in the process of a broadband surrogate model construction. In particular, the high-fidelity EM model of the given structure, ***R****_f_*, implemented in Sonnet ***em*** by using a 0.5 × 0.5 grid (in microns), thick metal model and fine meshing (cf. Section 2.2), takes approximately 64 min to return single-frequency simulation data. On the other hand, the coarse-discretization EM model (denoted as ***R****_cd_*) requires only 6 s to provide single-frequency results, being implemented on a coarser 2 × 2 grid (in microns), and using the thin metal model as well as coarse meshing.

The low-fidelity EM model is utilized to acquire a set of training samples for the setup of a surface response approximation model. The particular implementation of the data-driven model is based on the kriging interpolation as detailed below.

Let *X*_B_ = {***x***^1^, ***x***^2^, …, ***x****^N^*} denote a base set, such that the responses ***R****_cd_*(***x****^j^*) are known for *j* = 1, 2, …, *N*. Let ***R****_cd_*(***x***) = [*R_cd_*_.1_(***x***) … R*_cd.m_*(***x***)]*^T^*, where vector components correspond to complex admittance evaluated at *m* frequency points.

Here, ordinary kriging is adopted, where a deterministic function *f* is estimated as *f_p_*(***x***) = *µ* + *ε*(***x***), with *µ* denoting the mean of the response at base points, and *ε* being the error with zero expected value and a correlation structure depending on a generalized distance between the base points [31]. A Gaussian correlation function of the following form is used

(1) $$R(x^{i},x^{j})=exp[\sum_{k=1}^{N}\theta_{k}{\left|x_{k}^{i}-x_{k}^{j}\right|}^{2}]$$

where *θ_k_* are unknown hyper-parameters used to fit the model; *x_k_^i^* and *x_k_^j^* are the *k*th components of the base points ***x****^i^* and ***x****^j^*.

The kriging coarse model ***R****_c_* is defined as

(2) $$R_{c}(x)={\left[R_{c.1}(x)\;...\;R_{c.m}(x)\right]}^{T}$$

where


(3) $$R_{c.j}(x)={\bar{\mu }}_{j}+r^{T}(x)R^{-1}(f_{j}-1{\bar{\mu }}_{j})$$

In the above formulation, ***1*** denotes an *N*-vector of ones, ***f****_j_* = [*R_cd.j_*(***x***^1^) … *R_cd.j_*(***x****^N^*)]*^T^*, while ***r*** = [*R*(***x***,***x***^1^) … *R*(***x***,***x****^N^*)] is the correlation vector between the point ***x*** and base points, whereas ***R*** = [*R*(***x****^i^*,***x****^j^*)]*_i_*_,*j* = 1,…,*N*_, is the correlation matrix between the base points. The mean ${\bar{\mu }}_{j}$ is given by

(4) $${\bar{\mu }}_{j}={\left(1^{T}R^{-1}1\right)}^{-1}1^{T}R^{-1}f_{j}$$

Hyper-parameters *θ_k_* are found by maximizing $-[N\ln({\bar{\sigma }}^{2})+\ln|R|]/2$ , where |*R*| and the variance ${\bar{\sigma }}_{j}^{2}={\left(f_{j}-1{\bar{\mu }}_{j}\right)}^{T}R^{-1}(f_{j}-1{\bar{\mu }}_{j})/N$ are functions of *θ_k_* [31].

It should be noted that the kriging interpolation model given by (2) and (3) is merely a representation of the coarse-discretization EM model ***R****_cd_*. Thus, it requires refinement to become a reliable surrogate ***R***_s_, i.e., an accurate representation of the high-fidelity model ***R****_f_*. In the present work, this is accomplished by a combination of the input, frequency, and multiplicative output space mapping of the form

(5) $$R_{s}(x)=A\cdot R_{c}(B\cdot x+c;f_{1}+f_{2}\Omega )+d$$

In (5), ***R****_c_*(***x***;*f*_1_ + *f*_2_Ω) is the frequency scaled coarse model [32], where Ω is the frequency sweep, whereas ***F*** = [*f*_1_ *f*_2_]*^T^* are the scaling parameters of the affine transformation Ω ← *f*_1_ + *f*_2_Ω. The SM parameters are found by minimizing the expression ∑*_k_*_= 1,…,*NSM*_ ||***R****_f_*(***x****^k.SM^*) − ***A***⋅***R****_f_*(***B***⋅***x****^k.SM^* + ***c***; *f*_1_ *+ f*_2_Ω)|| w.r.t. the matrices ***A***, ***B***, and the vectors ***c*** and ***F***. The base set *X_B_*_.*SM*_ = {***x***^1.*SM*^, ***x***^2.*SM*^, …, ***x****^NSM.SM^*} is allocated using star distribution (2*n* + 1 samples with *n* being the number of design parameters) [32].

Observe that using kriging interpolation as a means of representing the coarse-discretization EM model ***R****_cd_* is not critical here and other types of approximation models could be implemented instead (e.g., artificial neural networks [33], support vector machines [34], rational functions [35], etc.). Most importantly, regardless of the data-driven modeling approach used here to create the coarse model ***R****_c_*, it becomes a reliable surrogate ***R****_s_* only after applying space mapping corrections as shown in (5).

### 3.3. Numerical Results

A broadband surrogate model is developed here for the integrated inductor shown in Figure 4 by using the methodology elaborated in Section 3.2. The feasible design space is defined by the lower/upper bounds ***l*** = [2 1 56]*^T^* μm, and ***u*** = [16 7 140]*^T^* μm. In the first step, the kriging model is set up using 480 ***R****_cd_* samples allocated on a uniform rectangular 8 × 4 × 15 grid. The space-mapping-corrected version of the low-fidelity kriging model ***R****_c_* is created as in (5) using 7 star-distributed ***R****_f_* samples. ***R****_cd_* simulation samples used for setting up the kriging model comprise the complex admittance *Y*_11_ in the frequency range from 0.1 GHz to 10 GHz, which can be subsequently recalculated to obtain the actual figures of interest, i.e., inductance *L* and the quality factor *Q* [36].

**Figure 6:**
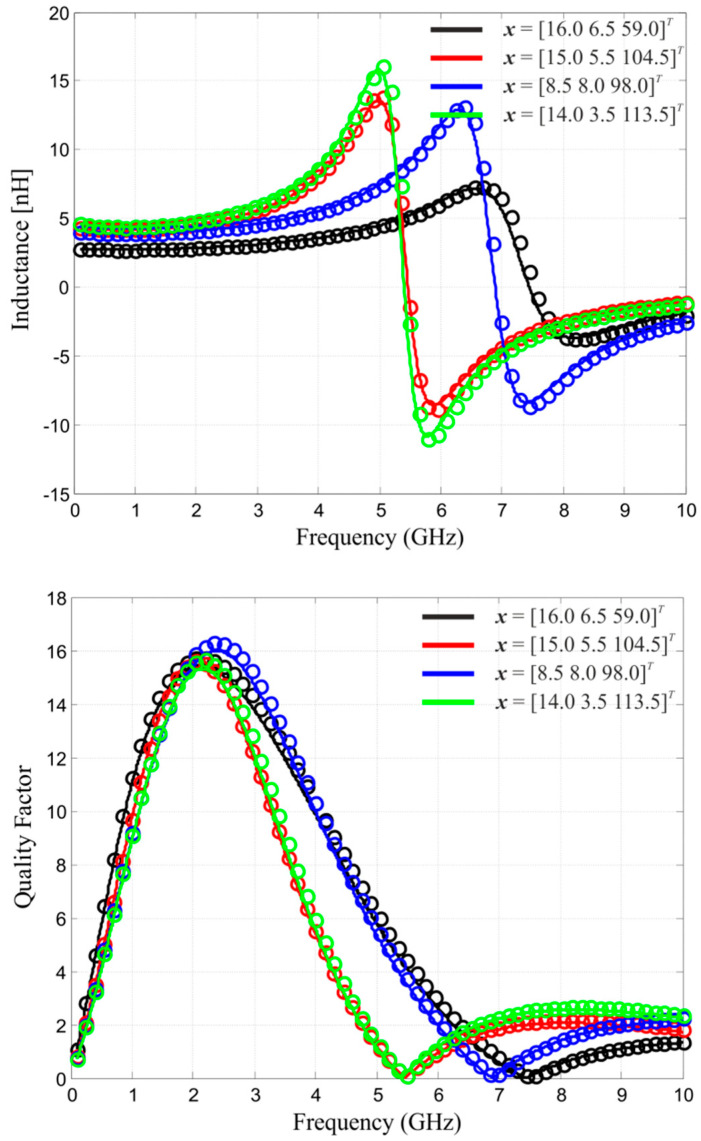
Frequency characteristics of the on-chip inductor at the selected test points: surrogate model ***R****_s_* (○) and high-fidelity EM model ***R****_f_* (-).

Frequency characteristics of *L* and *Q* at the selected test points for ***R****_s_* and ***R****_f_* are depicted in Figure 6. The relative RMS error acquired for 50 random test designs—for both the kriging model and the SM surrogate—is reported in Table 1. It can be observed that space mapping plays an extremely important role in improving the accuracy of the original kriging model. Additionally, the overall model accuracy is very good, especially considering its low setup cost (cf. Table 2). Note that each model evaluation typically requires six frequency points using adaptive band synthesis [26].

### 3.4. Inductor Design Optimization Application Example

The usefulness of the previously developed surrogate model is illustrated in this section based on inductor design optimization. The objective is to find a design solution that offers a specified inductance value *L*_target_ as well as maximizes the quality factor *Q*, both at a defined operating frequency *f*_0_. In addition, the peak frequency *f_Q_*_max_ (the frequency corresponding to the maximum quality factor) should be moved to *f*_0_ as close as possible, whereas the inductor layout area should be controlled by explicit constraints. It is also imperative that the final results are at the level of the high-fidelity model. With this in mind, the design problem can be formulated as

(6) $$\underset{x,A(x)\leq A_{\max}}{x_{f}^{*}=\mathrm{argmin}U(R_{f}(x))}$$

where ***R****_f_*(***x***) denotes the response vector of a high-fidelity EM model, ***x*** is a vector of designable parameters, *A*(***x***) is the circuit layout area, and *A*_max_ is a user-defined maximum acceptable layout area. The objective function is defined according to the above specification as follows

(7) $$U(R_{f}(x))=-Q(x)+\beta_{1}{\left[L(x)-L_{\mathrm{target}}/L_{\mathrm{target}}\right]}^{2}+\beta_{2}{\left[f_{Q\max}(x)-f_{0}/f_{0}\right]}^{2}$$

The objective function formulation used here gives full priority to the quality factor, while the successive penalty components contribute to the objective function only if the respective auxiliary performance conditions are violated. Thus, this allows for the maximization of the quality factor, while forcing *L* to *L*_target_ and *f_Q_*_max_ to *f_0_*. The penalty coefficients *β*_1_ and *β*_2_ are chosen so that the corresponding functions assume observable values (when compared to *Q*(***x***)) for relative violations larger than a few percent. For the given example, penalty coefficients are set to 1000, however, this specific value is not critical. In addition to the above, the penalty functions are continuous and differentiable with respect to the given performance figures, which is a major advantage from the optimization standpoint.

The direct solving of (6) is prohibitive due to the high cost of ***R****_f_*. For the sake of computational efficiency, an iterative SBO formulation can be utilized. In more detail, to account for the non-zero error of the surrogate model, the additive output space mapping algorithm [30] is applied to perform inductor optimization as given below:

(8) $$\underset{x,A(x)\leq A_{\max}}{x^{\left(i+1\right)}=\mathrm{argmin}U(R_{S}(x)+[R_{f}(x^{\left(i\right)})-R_{S}(x^{\left(i\right)})])}$$

For demonstration purposes, the process (8) was executed for the design case geared towards maximizing *Q* at *f*_0_ = 4.2 GHz, while obtaining *L*_target_ = 4 nH at *f*_0_. In addition, the process (8) is constrained by *A*_max_ = 28,000 μm^2^. The lower and upper bounds of the feasible design space ***lb***, ***ub***, the initial design ***x*^(^**^0)^ (set in the center of the domain) as well as the final design x* are summarized in Table 3.

**Figure 7:**
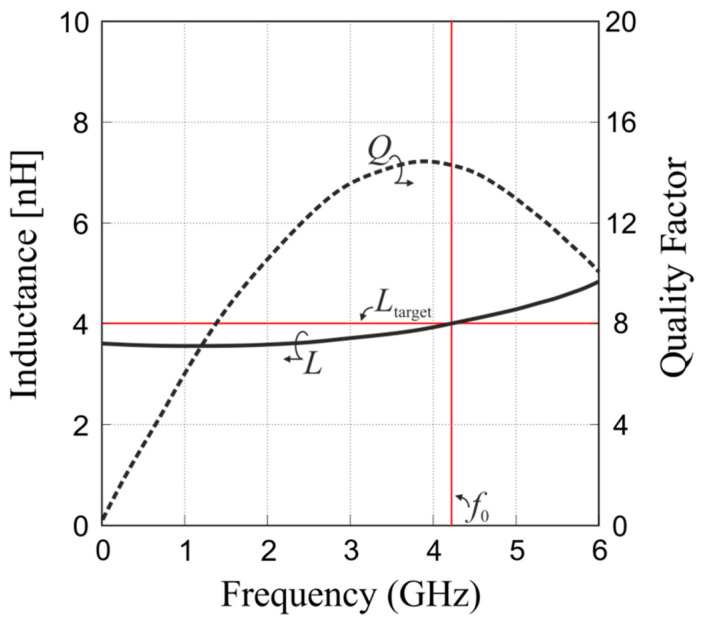
Responses of the compact integrated on-chip inductor at the initial (dashed, ---) and final (solid, -) design solutions. The horizontal and vertical red lines denote the target inductance and the operating frequency *f*_0_, respectively.

The operational frequency inductance obtained for ***x***^(0)^ relatively differs from *L*_target_ by about 35%, which is the reason for executing the optimization procedure. The final design x* was obtained in two iterations of (8), which means that only two high-fidelity EM simulations of the considered coil were conducted. The final inductor performance is shown in Figure 7. Table 4 provides detailed data on the structure of interest at the optimized design. One should note that the optimization process was restricted to the simulation grid of the high-fidelity EM model (i.e., 0.5 μm × 0.5 μm), which obviously limits the resolution of the design and does not allow for more precise satisfaction of the specifications or the quality factor improvement.

## 4. Multi-Fidelity Design Optimization of Compact On-Chip Inductors

This section presents a technique that allows for cost-efficient design optimization of integrated inductors. The proposed approach exploits EM simulations of variable fidelity, a pattern search optimization algorithm, and a design refinement procedure involving a local polynomial approximation model corrected by output space mapping. The optimum design can be reached at the cost of just a few high-fidelity EM simulations of the considered structure.

### 4.1. Design Case

Let us consider the design case of the integrated on-chip inductor of Figure 4. For the sake of simplicity, the multi-fidelity optimization framework is showcased here using the same structure, parameterization and design problem formulation as in the previous design example (cf. Section 3.1 and Section 3.4), yet different specifications.

### 4.2. Multi-Fidelity Optimization Algorithm

A standard approach to design small-size integrated inductors based on optimization can be formally expressed as a minimization problem of the form (6). Regardless of the specific optimization algorithm, this process requires multiple objective function calls, which are typically intractable due to excessive numerical cost related to a high-fidelity EM simulation involved in objective function evaluation.

To alleviate this fundamental obstacle, the technique under discussion adapts the multi-fidelity optimization algorithm [37] that exploits a family of coarse-discretization EM models of increasing fidelity {***R****_c.j_*}, *j* = 1, …, *K*, where ***R****_c.j_*_+1_ is finer than ***R****_c.j_*, which translates into better accuracy, but also a longer processing time. As opposed to lumped-element equivalent circuits that are currently widely used for design optimization of planar inductors, variable-fidelity EM models offer relatively good prediction and generalization capabilities [38]. In this work, all {***R****_c.j_*} models are implemented and evaluated using Sonnet (see Section 2.2 for details on the definition of a low-fidelity EM model).

The multi-fidelity algorithm operates in the following manner. Starting from the initial design ***x***^(0)^, the coarse-discretization EM model of the lowest fidelity, ***R****_c._*_1_, is optimized to arrive at ***x***^(1)^, which represents the first approximation of the high-fidelity model optimum ***x***_f_*. Having obtained ***x***^(1)^, it is subsequently used as the starting point for optimization of another model in the line to produce an even more accurate approximation of ***x***_f_*. This is repeated in a sequence until the optimum ***x***^(*K*)^ of the finest among coarse-discretization EM models, ***R****_c.K_*, is found. Normally, only a few ***R****_c_* models are needed for fast convergence of the algorithm under discussion. In this work, the process can be effectively accomplished with *K* = 2. Additionally, a pattern search algorithm [39] is used here to optimize all coarse-discretization EM models. Having obtained the optimized design x^(*K*)^ of the finest coarse-discretization model ***R****_c.K_*, it is evaluated at perturbed designs around ***x***^(*K*)^, i.e., at ***x****_k_*^(*K*)^ = [*x*_1_^(*K*)^ … *x*_k_^(*K*)^ + sign(k)·*d_k_* … *x_n_*^(*K*)^]*^T^*, *k* = −*n*, −*n*+1, …, *n* − 1, *n*. Notation ***R***^(*k*)^ = ***R****_c.K_*(***x****_k_*^(*K*)^) is used here. These data are applied to refine the finest approximate design without directly optimizing ***R****_f_*. The latter is omitted by setting up an approximation model based on ***R***^(*k*)^, and optimizing it in the neighborhood of ***x***^(*K*)^ defined as [***x***^(*K*)^ − ***d***, ***x***^(*K*)^ + ***d***], where ***d*** = [*d*_1_ *d*_2_ … *d_n_*]*^T^*. The approximation is performed using a reduced quadratic model *q*(***x***) = [*q*_1_ *q*_2_ … *q_m_*]*^T^*, defined as [40]

(9) $$q_{j}(x)=q_{j}({\left[x_{1}\ldots x_{n}\right]}^{T})=\lambda_{j.0}+\lambda_{j.1}x_{1}+\ldots +\lambda_{j.n}x_{n}+\lambda_{j.n+1}x_{1}^{2}+\ldots +\lambda_{j.2n}x_{n}^{2}$$

Coefficients *λ_j.r_* can be found analytically [37] by solving a system of linear regression problems of the form *q_j_*(***x****_k_*^(*K*)^)  =  ***R****_c.K_*(***x****_k_*^(*K*)^) for *k*  =  −*n*, …, 0, …, *n*. In the final step, the refined design is found as:

(10) $$\underset{x^{\left(K\right)}-d\leq x\leq x^{\left(K\right)}+d,\;A(x)\leq A_{\max}}{x^{*}=\mathrm{argmin}U(q(x)+\Delta q(x^{\left(K\right)}))}$$

where the output space mapping term Δ***q***(***x***^(*K*)^) = ***R****_f_*(***x***^(*K*)^) − ***R****_c.K_*(***x***^(*K*)^) accounts for misalignment between ***R****_c.K_* and ***R****_f_* [37]. The optional frequency scaling of ***q*** is also possible to account for frequency shift between ***R****_c.K_* and ***R****_f_* [41]. If necessary, step (10) can be repeated starting from a refined design, i.e., x* = argmin{***x***^(*K*)^ − ***d*** ≤ ***x*** ≤ ***x***^(*K*)^ + ***d***: *U*(***q***(***x***) + Δ***q***(x*) (constraint placed on *A*(***x***) is omitted here for the sake of clarity). Note that each iteration requires only one evaluation of the high-fidelity EM model ***R****_f_*.

### 4.3. Numerical Results

This section presents numerical data acquired in the process of multi-fidelity design optimization of the integrated inductor shown in Figure 4. The high-fidelity EM model ***R****_f_* was set up as described in Section 3.2. In addition, two coarse-discretization EM models—***R****_c_*_1_ and ***R****_c_*_2_—were defined by using 2 μm × 2 μm and 1 μm × 1 μm grids, respectively, as well as the thin metal model and coarse meshing. Their respective simulation times are 60 s and 150 s.

The presented procedure is aimed at maximizing *Q* at *f*_0_ = 3.25 GHz while reaching *L*_target_ = 3.49 nH at the same frequency. An additional objective is to keep the inductor’s layout area below or equal to *A*_max_ = 13,000 μm^2^. The bounds of the search space, as well as the starting point for the optimization process ***x***^(0)^, are collected in Table 5. The initial design exhibits an inductance of 4.55 nH at the operating frequency, which is relatively 30% off from the target value of 3.49 nH.

The optimized design x* reaches the specified inductance at the operating frequency and the quality factor of 14.1 at *f*_0_. This result was obtained at the total cost of six evaluations of ***R****_f_* (including three iterations of the refinement procedure (10), cf. Table 6 for a detailed cost breakdown). The cost-efficiency of the presented method can be highlighted when compared against alternative design routines that include: (i) direct derivative-free optimization of the ***R****_f_* model using a pattern search algorithm [39], (ii) exhaustive enumeration, (iii) customized enumeration and (iv) implicit space mapping with geometric programming [19]. The results obtained by the presented method as well as alternative approaches (i–iv) listed above are compared in Table 7. The first alternative approach considered in the comparison yields the same final design as the one obtained by the method presented in Section 4, x*_(i)_ = x*. However, this solution is identified at the cost of 42 evaluations of the high-fidelity model ***R****_f_*, which demonstrates a 7-fold speedup of the presented method when compared to a standard EM-driven optimization routine of [39]. In exhaustive enumeration, sampling of the domain using 2 μm, 1 μm, and 0.5 μm step sizes for every design dimension translates into 1376, 8925, and 63,713 EM model simulations, which is clearly intractable in a reasonable time frame, yet guarantees finding the actual discreet optimum. In the customized enumeration of (iii), developed for comparison purposes, the process begins with selecting a pool of candidate solutions from the complete set of designs obtained by sampling the search space with a medium step of 1 μm; the candidate solutions are chosen based on their footprint area subject to a user-defined constraint of ≤ Amax and ≥ *γ*%·Amax (here, *γ* = 99) and are subsequently simulated using ***R****_c_*_2_ coarse-discretization EM model. The candidate pool is further narrowed down by selecting those designs whose inductance satisfies *L*_target_ ± δ*_L_*_target_, where deviation *δ*_Ltarget_ is picked by the user to account for EM model inaccuracy (here, *δ*_Ltarget_ = 15%). Finally, the pool of candidate solutions is expanded iteratively by producing new designs in the vicinity of the base solutions by changing every single parameter by ±0.5 μm, with designs that violate *A*_max_ constraint or lower/upper bounds being discarded from the pool. The EM-verification of the first-iteration candidate pool requires 18 ***R****_f_* simulations, whereas the next one involves additional 59 ***R****_f_* simulations. This corresponds to the cost of finding the first-iteration optimum ***x***^(1)^_(iii)_ and the second-iteration optimum ***x***^(2)^_(iii)_, respectively. The last method included here as a benchmark is an implicit space mapping (ISM) algorithm with an approximate circuit-model-based geometric programming objective function formulation of [19]. The ISM approach produces a satisfactory design x*_(iv)_ at the cost of eight EM simulations, which is the second most efficient way of reaching a comparable design solution included in method comparison (cf. Table 7).

**Figure 8:**
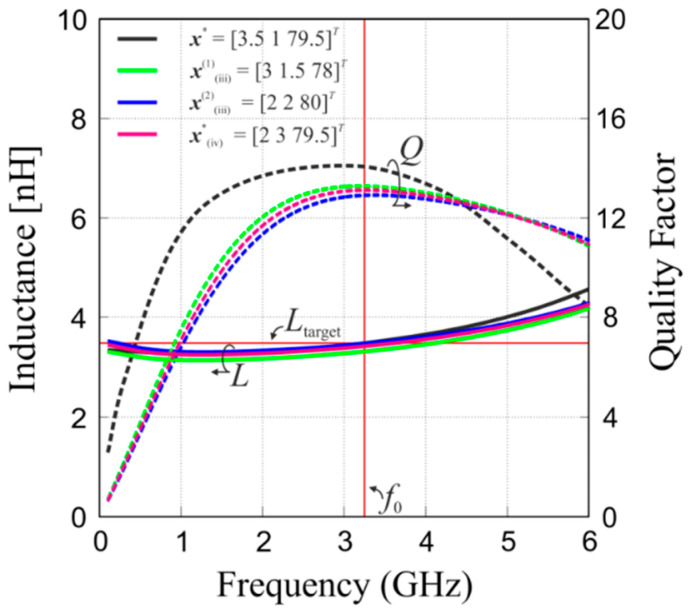
Frequency characteristics of the on-chip inductors designed by different methods: this work (black), 1st iteration of customized enumeration (green), 2nd iteration of customized enumeration (blue), ISM (violet). Horizontal and vertical solid red lines denote the target inductance and the operating frequency, respectively.

The EM-simulated frequency characteristics of the final inductor designs obtained by using the proposed method and alternative approaches are given in Figure 8. Inspection of Table 7 and Figure 8 reveals that the design solutions delivered by the proposed method (as well as the pattern search algorithm of (i)) best satisfy design specifications and offer the highest *Q*-factor at the operating frequency. In terms of computational efficiency, the proposed method is superior to standard EM-driven routines (i–iii) by at least a couple of times, whereas the SBO benchmark technique of (iv) is only 33% more CPU expensive, but is also less convenient to use as it requires setting up both EM and circuit-based inductor models.

The specific inductor design solution obtained in this section can also be set against previously reported designs from the literature under the condition that the respective design specifications are a close match. To this end, Table 8 compares several designs in terms of their key figures of merit and layout area. One can observe that—even though the specified inductance value at the operating frequency is met—the results differ in terms of the number of turns, the quality factor as well as inductor layout area. We can also observe that the results can be placed in an order, meaning that the higher the operating frequency value of the quality factor, the larger the inductor footprint area. This general conclusion reveals that the quality factor maximization and inductor footprint area minimization are in fact conflicting design objectives and that the design problem of compact inductors as defined in this work is merely a specific case of a more general, multi-objective problem formulation. From this perspective, the results presented in Table 8 belong to the Pareto-optimal set of solutions, which contains designs that are optimal in a multi-objective sense, meaning that no better solution can be found that improves one objective without worsening the other one [42]. Of course, approaching the design problem from the multi-objective standpoint is much more complex than solving the problem in a single-objective setting as it predominantly requires population-based optimization algorithms [42].

## 5. Discussion and Conclusions

This work discusses the cost-efficient design of compact integrated on-chip inductors by means of surrogate-based optimization and EM simulation models of variable fidelity. The presented methodologies are more generic and provide more accurate results than the previously reported design techniques, largely based on inaccurate lumped-element equivalent circuits. At the same time, they illustrate computational superiority over conventional optimization routines resorting only to high-fidelity EM models.

The main benefit of applying the presented methods is in using the available computational resources and software licenses much more efficiently than by resorting merely to standard options/techniques available commercially, which results in higher productivity and output. Apart from the verification inductor example provided in this work (cf. structure of Figure 4), the proposed methodologies can be successfully (and directly) used for any other type of inductor, regardless of its geometry, design requirements or technology process. This is because the surrogate-based optimization techniques presented here are independent of these model-related factors. Moreover, with small adjustments made to the objective function formulation, the demonstrated methods could also be applied to other types of components or circuits in microwave technology and related fields. In fact, a substantial shortening of simulation-driven design cycles of complex and computationally expensive devices and systems can be of great practical interest for engineers across different specializations ranging from microwave/RF engineering to aerospace or mechanical engineering [45].

It should be reiterated that the computational efficiency, as well as the convergence rate of the surrogate-based optimization methods, are strictly associated with the CPU cost and accuracy of the low-fidelity models. The selection of a suitable low-fidelity model that minimizes the total numerical cost of the surrogate-based optimization process is a major issue that to this date remains open. The future work of the author will focus on handling this problem.

## Figures and Tables

**Table 1:** On-chip inductor surrogate model accuracy.

Model	Relative RMS Error [%]	
	Inductance *L*	Quality Factor *Q*
***R*** *_c_* ^1^	17.0	15.3
***R*** *_s_* ^2^	10.5	6.8

^1^ Coarse model developed using kriging interpolation based on coarse-mesh simulation data. ^2^ Surrogate model developed by correcting ***R***_c_ using space mapping technology.

**Table 2:** On-chip inductor surrogate model setup cost.

Modeling Stage	Number of Model Evaluations	Computational Cost	
		Absolute [h]	Relative to *R_f_*
Kriging Coarse Model	480 × ***R****_cd_*	4.8	0.75
Space Mapping Corrections	7 × ***R****_f_*	44.8	7
Total cost	n/a	49.6	7.75

**Table 3:** Design example no. 1 summary: ***lb***, ***ub*, *x***^(0)^, and x*.

	*x*_1_ [μm]	*x*_2_ [μm]	*x*_3_ [μm]
Lower bounds: ***lb***	2	1	56
Upper bounds: ***ub***	16	7	140
Initial design: ***x*****^(0)^**	9.0	4.0	98.0
Final design: x*	7.5	3.5	86.0

**Table 4:** On-chip inductor optimization results.

Design Specifications		*L*, *Q*, and Maximum-*Q* Frequency at the Final Design		
*L*_target_	*f*_0_	*L*	*Q*	*f_Q_*_max_
4 nH	4.2 GHz	4 nH	14.5	3.85 GHz

**Table 5:** Problem setup for design example no. 2: ***lb***, ***ub*,** and ***x***^(0)^.

	*x*_1_ [μm]	*x*_2_ [μm]	*x*_3_ [μm]
Lower bounds: ***lb***	2	1	56
Upper bounds: ***ub***	16	7	140
Initial design: ***x***^(0)^	9.0	4.0	98.0

**Table 6:** On-chip inductor optimization cost.

**Algorithm Step**	**Number of Model Evaluations**	**Computational Cost**	
		**Absolute [min]**	**Relative to** ***R**_f_*
Optimization of ***R****_c_*_.1_	20 × ***R****_c._*_1_	20	0.31
Optimization of ***R****_c_*_.2_	22 × ***R****_c._*_2_	55	0.86
Setup of model ***q***^1^	3 × 7 × ***R****_c._*_2_	53	0.83
Evaluation of ***R****_f_*	4 × ***R****_f_*	256	4
Total cost	n/a	384	6

^1^ ***q*** was set up three times (one for each refinement iteration).

**Table 7:** Comparison of different optimization techniques.

**Method**	**Final Design**	*L @ f*_0_	*Q @ f*_0_	*f*_Qmax_	**Layout Area**	**CPU Cost** **(Relative to *R***_*f*_ **)**
This work	x* = [3.5 1 79.5]*^T^*	3.49 nH	14.1	3.0 GHz	12,882 μm^2^	6
Pattern search algorithm	x*_(i)_ = [3.5 1 79.5]*^T^*	3.49 nH	14.1	3.0 GHz	12,882 μm^2^	42
Customized Enumeration ^1^	***x***^(1)^_(iii)_ = [3 1.5 78]*^T^*	3.32 nH	13.3	3.2 GHz	12,321 μm^2^	18.78
	***x***^(2)^_(iii)_ = [2 2 80]*^T^*	3.48 nH	12.9	3.37 GHz	11,664 μm^2^	77.78
ISM with GP	x*_(iv)_ = [2 3 79.5]*^T^*	3.44 nH	12.8	3.44 GHz	12,882 μm^2^	8

^1^ Exhaustive enumeration of (ii) was not executed due to excessive CPU cost; for the same reason, customized enumeration was terminated after the second iteration.

**Table 8:** Comparison of different design solutions.

**Ref.**	**Number of Turns**	**Specifications**			**Inductor Performance and Area**		
		*L*_target_	*f*_0_	*A*_max_	*L* @ *f*_0_	*Q* @ *f*_0_	Layout Area
[43]	3 ^1^	3.49 nH	3.25 GHz	–	3.49 nH	16.6	37,488 μm^2^
[44]	3.5 ^2^	3.50 nH	3.25 GHz	–	3.50 nH	16.3	30,002 μm^2^
[43]	4 ^1^	3.49 nH	3.25 GHz	–	3.50 nH	15.7	27,001 μm^2^
This work	4 ^1^	3.49 nH	3.25 GHz	13,000 μm^2^	3.49 nH	14.1	12,882 μm^2^
[43]	5 ^1^	3.49 nH	3.25 GHz	–	3.50 nH	12.5	6989 μm^2^

^1^ 1-layer rectangular spiral inductor in a differential configuration. ^2^ 1-layer rectangular spiral inductor in a single-ended configuration.
